# Online water quality monitoring based on UV–Vis spectrometry and artificial neural networks in a river confluence near Sherfield-on-Loddon

**DOI:** 10.1007/s10661-022-10118-4

**Published:** 2022-08-03

**Authors:** Hongming Zhang, Lifu Zhang, Sa Wang, LinShan Zhang

**Affiliations:** Aerospace Information Research Institute, Chinese Academy of Sciences, Beijing, 100101 China

**Keywords:** UV–Vis spectrophotometry, Convolutional neural networks, Turbidity compensation, Total organic carbon, Total suspended solids

## Abstract

Water quality monitoring is very important in agricultural catchments. UV–Vis spectrometry is widely used in place of traditional analytical methods because it is cost effective and fast and there is no chemical waste. In recent years, artificial neural networks have been extensively studied and used in various areas. In this study, we plan to simplify water quality monitoring with UV–Vis spectrometry and artificial neural networks. Samples were collected and immediately taken back to a laboratory for analysis. The absorption spectra of the water sample were acquired within a wavelength range from 200 to 800 nm. Convolutional neural network (CNN) and partial least squares (PLS) methods are used to calculate water parameters and obtain accurate results. The experimental results of this study show that both PLS and CNN methods may obtain an accurate result: linear correlation coefficient (R^2^) between predicted value and true values of TOC concentrations is 0.927 with PLS model and 0.953 with CNN model, R^2^ between predicted value and true values of TSS concentrations is 0.827 with PLS model and 0.915 with CNN model. CNN method may obtain a better linear correlation coefficient (R^2^) even with small number of samples and can be used for online water quality monitoring combined with UV–Vis spectrometry in agricultural catchment.

## Introduction

Water is a key natural resource for the maintenance of all ecosystems on the planet. Although 71 percent of the Earth's surface is covered by water, it remains a precious resource (Baker et al., 2016; El Habr, 1995). Because oceans hold approximately 96.5% of all Earth's water and fresh water accounts for only 1% of the Earth’s available water resources (Boberg, 2005), the conservation of water resources is critical to the sustainable development of all human beings (Falkenmark, 2020). In fact, studies have reported an increase in pollution derived from all kinds of production and operation activities, such as industrial, agricultural and sanitary sewage pursuits (Khalid et al., 2018). Rivers carry large quantities of water from the land to the ocean, forming part of the water cycle (Chahine, 1992). At the same time, the pollution of surface water with excessive nutrients and toxic substances is also occurring worldwide. One of the reasons for pollution events is world population growth, which has resulted in an increasing need for agricultural farming and urban activities. There is a need to monitor water quality continuously in the river confluences of agricultural catchments. At present, both chemical and physical methods are used to analyze water parameters. Chemical methods are stable and accurate but have some disadvantages, including long measuring periods and the generation of secondary pollution (Ma et al., 2020). Ultraviolet–visible (UV–Vis) spectrometry is a widely used physical method to detect or monitor water parameter quality (Avagyan et al., 2014; Hu et al., 2016). It is based on a regression model between a spectrum curve and parameters measured by chemical methods. Langergraber’s group developed UV–Vis spectrometry to measure in situ water quality parameters in real time (Langergraber et al., 2003; van den Broeke et al., 2006). The Mie scattering turbidity compensation method is used to calculate the dissolved particle distribution and reduce the influence of the measured in situ absorption spectrum (Chen et al., 2021). Multivariate statistical methods have been used in this context to predict water quality metrics such as total organic carbon (TOC), chemical oxygen demand (COD), total suspended solids (TSS) and nitrate levels. TOC is a measure of the total amount of carbon in pure water or aqueous systems and is used to monitor overall levels of organic compounds. In many situations, TOC is used as a monitor of organic content changes. TOC is traditionally measured by oxidizing the organic compounds in water to forms that can be measured. The CO2 generated is traditionally measured by the conductivity change of pure water after dissolving the above CO2. The conventional monitoring method is time consuming and expensive, including measurement processes and device maintenance costs. Total suspended solids (TSS) are particles that are larger than 2 µm in the water. Particles smaller than 2 µm are considered dissolved solids (Butler & Ford, 2018).

Bow Brook (a headwater tributary to the River Loddon) runs through a predominantly agricultural catchment from Pamber End to Sherfield-on-Loddon in Hampshire. However, the water quality of the agricultural catchment has not received effective monitoring. With a UV–Vis spectrometry water probe, water quality can be effectively monitored in real time. Thus, monitoring and sampling equipment was placed close to the confluence with the river Loddon near Sherfield-on-Loddon.

Multivariate statistics methods are typically used to analyze complex datasets that univariate analysis methods may properly address. In a practical application, multivariate statistical methods may involve several multivariate analysis models to build the relationship between variables and the parameters being studied (Chen et al., 2017). Partial least squares (PLS) regression methods have advantages over more traditional methods. PLS is a technique that reduces original data to a small set of uncorrelated components with which to perform least squares regression (Boulesteix & Strimmer, 2006). The PLS technique is useful when traditional regression methods fail or produce high standard errors. Even though it is typically used in drug, chemical and food industry applications to model the relationship between spectra and physicochemical properties, the PLS regression method is also used to measure COD, TOC, TSS and nitrates in just a single measurement.

In the last decade, artificial intelligence has witnessed great advances in development, technology and applications. Technology has had a great impact in almost every field that humans have dominated in the past (Esteva et al., 2021). Artificial neural network (ANNs) methods use mathematical computation to simulate the human brain process and has been applied in many fields to bridge the gap between human abilities and the external world. Artificial neural networks (ANNs) can implement a variety of complex nonlinear mappings with powerful pattern recognition and data fitting capabilities. One of the attempts of this field is to enable machines to perceive and analyze the world as humans would. A convolutional neural network is a feedforward neural network model that has shown good performance in image processing, image classification and feature extraction (Liu, 2018). Due to its feature extraction capability, the CNN model can perform classification and regression tasks using high-dimensional original raw data (Hu et al., 2015). Weight sharing and local perception may tremendously reduce the number of parameters and improve the learning rate of a network model (Véstias, 2019). Recently, CNNs have been used to predict water level and water quality as well as quantify cyanobacteria.

In this study, TOC and TSS in the river confluence near Sherfield-on-Loddon were monitored with UV–Vis spectrometry and different calibration models, requiring no chemical pretreatment or thermal reaction. With multiple scattering correction (MSC) and the SNV preprocessing method, the CNN model shows outstanding performance in calculating TOC and TSS water parameters.

## Materials and methods

### Study area and sample collection

**Fig. 1:**
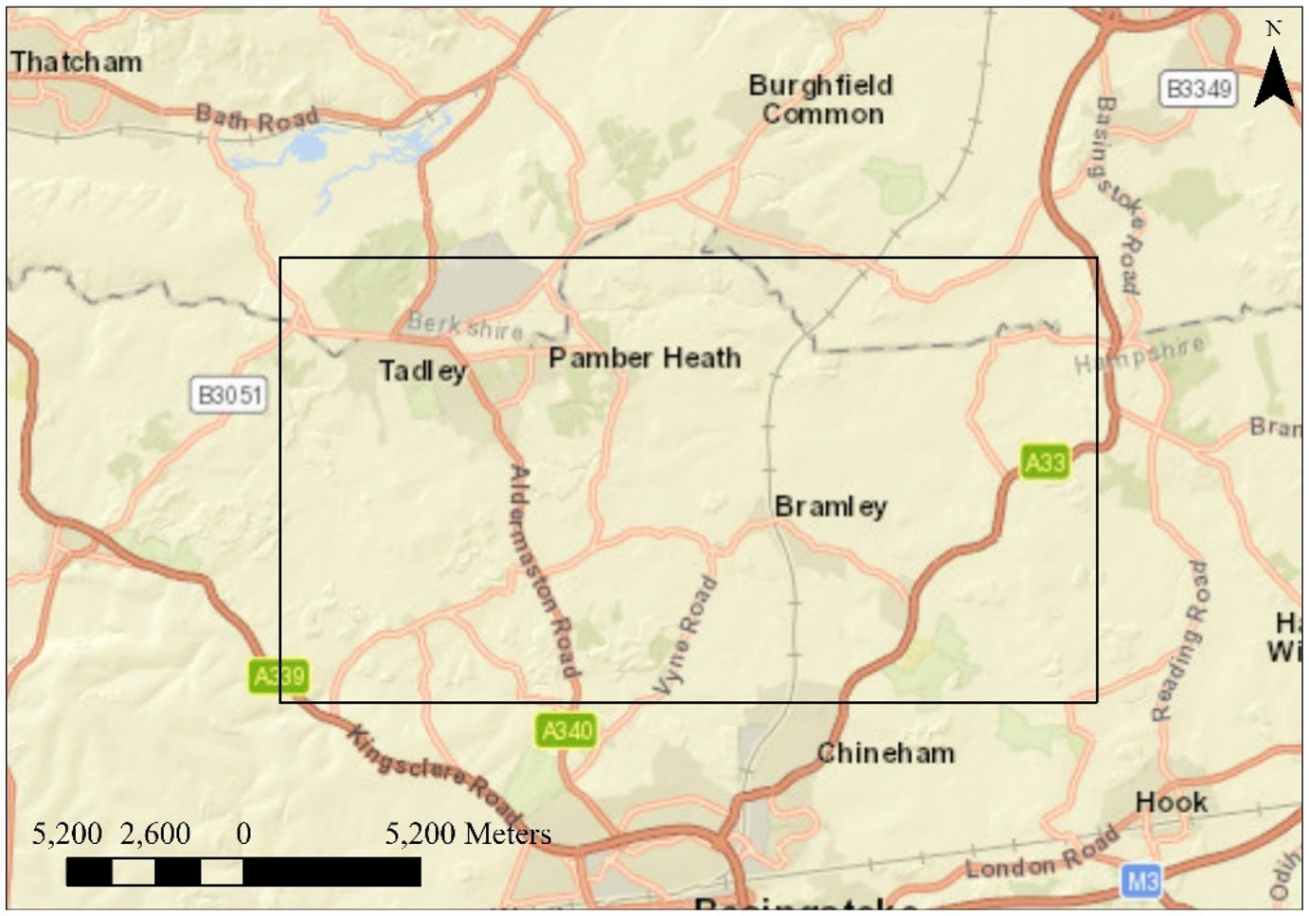
Study area

Figure 1 shows the location of the study area. Bow Brook (a headwater tributary to the River Loddon) runs through a predominantly agricultural catchment from Pamber End to Sherfield-on-Loddon in Hampshire. The monitoring and sampling equipment was placed close to the confluence with the Loddon River near Sherfield-on-Loddon (Hawkins et al., 2019). All daily samples were taken using two ISCO 6712 autosamplers at 9 am GMT from December 12, 2017, to April 2, 2018. Several samples are missing due to equipment breakdown because of temperature (frozen equipment). In this study, 94 samples are used for model construction. Samples were stored in a refrigerator at 4 °C on return to the laboratory and filtered within 48 h of collection. All samples were analyzed in the laboratory for a full spectral scan using a Jenway 7315 spectrophotometer in the laboratory before and after filtering by 0.7-µm filters. Before sample measurement, a baseline scan was performed to reduce the background to zero due to the absorption of cuvettes and water. The cuvette was rinsed twice with deionized water and once with the sample before the actual spectral measurement. For low organic content and large inorganic content water, the NPOC method was chosen to avoid possible negative results that may come from subtraction of total carbon (TC) –total inorganic carbon (TIC). Ten milliliters of filtered sample solution was acidified using 0.1 ml of 15% v/v HCl to give a pH between 2 and 3. Approximately 7 to 8 ml of the acidified sample was transferred to a 9-ml glass vial and used for NPOC on a Shimadzu TOC-L, and the top was covered with aluminum foil.

### Model construction for TOC and TSS calculation

**Fig. 2:**
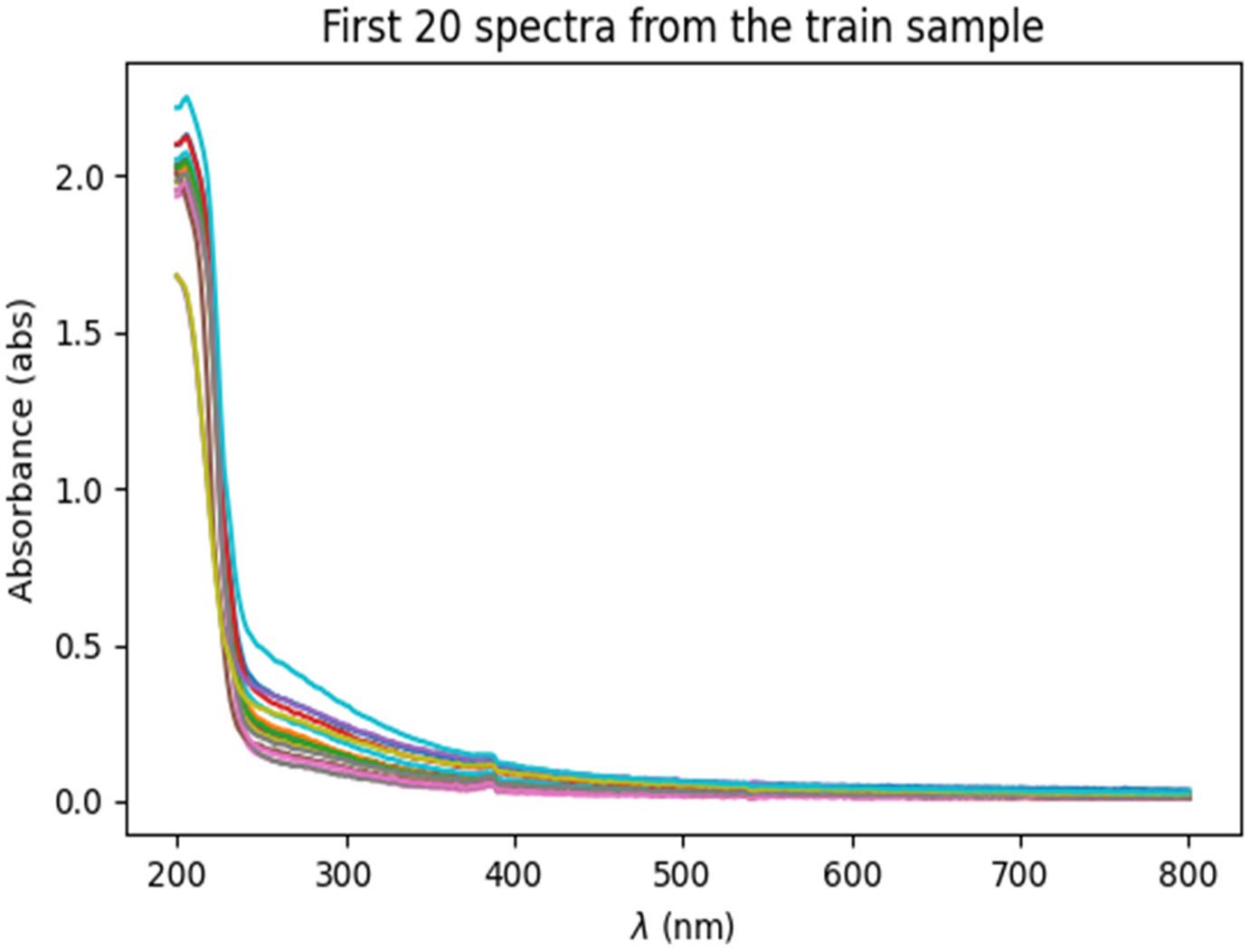
UV–Visible absorption spectra of the first 20 training samples

Turbidity is the main indicator of rainwater runoff pollution, which can also decrease the spectrum shape feature. Multiple scattering correction (MSC) and standard normal variate (SVN) methods are used to process the raw UV–Vis absorption spectra to reduce the influence of turbidity particle scattering and enhance the spectral absorption information related to the water content of components. Figure 2 shows the UV–Vis absorption spectra of the water sample. Two methods are used to construct the model with totally 94 samples (80% dataset as training data, 20% dataset as test data), including partial least squares (PLS) regression and convolutional neural networks (CNNs). These two methods are compared using the same daily collected water sample data. This is a statistical method used to develop a regression model of predicted values and measured values based on covariance. The PLS regression method reveals its usefulness with its ability to analyze data with noisy, collinear and even incomplete variables in both X and Y (Wagala et al., 2020). With the increasing number of variables and results, the accuracy of the model will improve. CNNs are artificial neural networks that are usually composed of several convolutional layers. CNNs are dominant in various computer vision applications, such as facial recognition, target detection, image recognition, image annotation, image theme generation, image content generation and object annotation. However, CNNs are also used for data regression analysis (Jernelv et al., 2020). One-dimensional spectral data may be reshaped to two-dimensional arrays, and a 2D CNN model can be used for regression applications. Another method is to use a one-dimensional CNN in the regression method. In this study, we use a one-dimensional convolutional network and handle the sample according to the input data. A CNN network model can perform both linear and nonlinear regression.

**Fig. 3:**
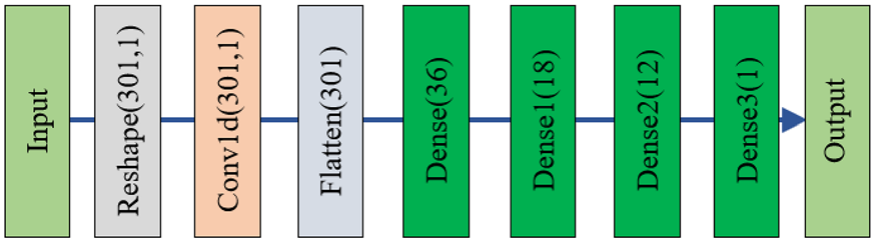
Schematic of the CNN network

We apply a one-dimensional convolutional network and reshape the input data according to it. Figure 3 shows the schematic overview of the CNN model. It consists of a convolutional layer, a pooling layer and a fully connected layer. The spectral data are converted into 301*1 one-dimensional spectral vectors after processing with the MSC and SVN models. The first convolutional block has two convolution kernels to extract spectral features and one pooling layer to reduce the number of model parameters while maintaining the feature information. Convolutions are performed through a convolutional layer with a window width of 5. The continuous dense performance reduces the noise and dimensional size of the spectrum. The regression operation is performed with a fully connected layer, and the output is the result.

Many evaluation criteria have been used in previous studies to select the best performance regression model. The criteria accuracy can be influenced by the sample concentration range and sample number. Samples can be classified into several classes according to the sample concentration range, and different models are used in each range. In this study, root-mean-square error (RMSE) and R-squared (R^2^) are used to evaluate the quality of the regression model. The root-mean-square error (RMSE) is a standard way to evaluate the error of a model in predicting quantitative data (Nabavi-Pelesaraei et al., 2021). R-squared (R^2^) is a statistical measure of fit that represents how much variation of a dependent variable is explained by the independent variable or variables in a regression model (Alexopoulos, 2010). R^2^ should be as close to 1 as possible. If R^2^ is above 0.9, the model performs well. If R^2^ is between 0.7 and 0.9, the model is fairly accurate (McNeil & Chilvers, 2000). If R^2^ is below 0.7, the model is poor and should only be used for qualitative analysis. In Eqs. (1) and (2), n is the number of sample data points, ${\text{y}}_{i}$ is the observed value, $f_{i}$ is the predicted value, and $\mathop y\limits^{ - }$ is the averaged value of the observed value.

1 $$RMSE=\sqrt{\sum_{i=1}^{\mathrm{n}}\frac{\left({f}_{i}-{y}_{i}\right)}{n}}$$

2 $${R}^{2}=1-\frac{{ss}_{res}}{{SS}_{tot}}=\frac{\sum_{i}{\left({y}_{i}-{f}_{i}\right)}^{2}}{\sum_{i}{\left({y}_{i}-\overline{y }\right)}^{2}}$$

Samples were randomly divided into a training set and a test set at a 9:1 ratio. The training dataset was used to train the model. In each training cycle, the training learning rate was set to 0.01, the number of batches was 16, and the number of iterations was 1000. The model weights and offsets were initialized randomly. The root-mean-square error (RMSE) was used as the loss function of the model. RMSE is the most commonly used regression loss function that measures the sum of squared distance between predicted values and training data values (Schmidt et al., 2019). The purpose of the training was to find the optimal threshold that minimizes the predicted values from the true values. The Adam optimization algorithm is an extension to stochastic gradient descent that is not affected by the expansion and contraction of the gradient and can be used to update model weights and offsets in the training data (Vasudevan, 2020). The RMSE loss function behaves well even for small loss values and will converge even with a fixed learning rate. The gradient of RMSE is varied, which means that RMSE loss is high when loss function values are larger and decreases as the loss function approaches 0, thus making RMSE more precise at the end of model training. After the training process, test data are used to evaluate the training efficiency of the model. Test data are not used in the training process to avoid overfitting.

## Results and discussion

### Performance of the constructed models

According to the Lambert–Beer law, the concentration of some components is linearly related to the absorption of incident light (Mayerhöfer et al., 2020). A linear model would be sufficient according to accuracy and robustness. Absorption spectroscopy interfaces with the turbidity of the water sample, which reduces the model accuracy (Wu et al., 2019). A nonlinear model would be more effective. For a CNN network model, a multilayered network may express the relationship of absorption spectroscopy and water parameters with linear and nonlinear patterns. The online monitoring method may provide a large amount of data that is needed to train the neural network. In practice, constructing the model needs to take mathematical difficulty and accuracy into account. A single model may not handle all different kinds of water types. For each type of water sample, constructing a specific model may provide efficient accuracy and robustness (Ta & Wei, 2018). In this study, PLS and CNN methods were used to estimate the TOC and TSS of the sample at river confluence with UV–Vis spectroscopy and RMSE and R^2^ as the criteria.

**Fig. 4:**
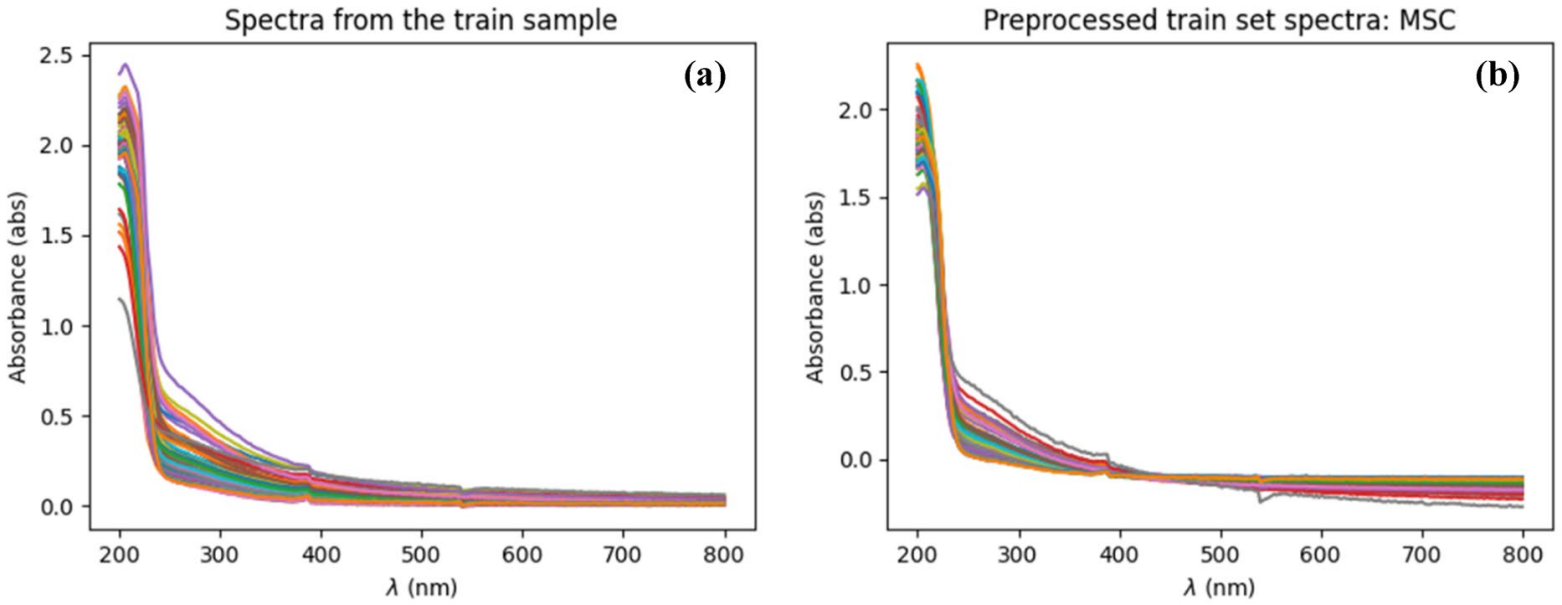
Turbidity compensation with MSC method

Even with multiwavelength method, the accuracy of inversion algorithm of water parameter is still difficult to reach a satisfactory level because of turbidity interference. Various turbidity compensation methods have developed to decrease or nearly eliminate the turbidity interference of suspended particles to water components. The multiplicative scatter correction (MSC) method is used in this study to correct the interference effect of spectra by particles in water samples. The MSC method corrects the spectrum by changing the scale and offset with the reference spectrum, which is the average spectrum of the samples. The MSC method needs a large number of samples to achieve a reasonable average spectrum to obtain a better compensation effect (Rinnan et al., 2009). Other studies use absorption spectrum of Double-Distilled Water as the reference spectrum in the MSC model. Figure 4 shows the spectrum before and after the MSC compensation operation. The compensated spectrum is smaller than the original spectrum because of the subtraction of offset and scale change, which is thought to be the interference of turbidity.

**Fig. 5:**
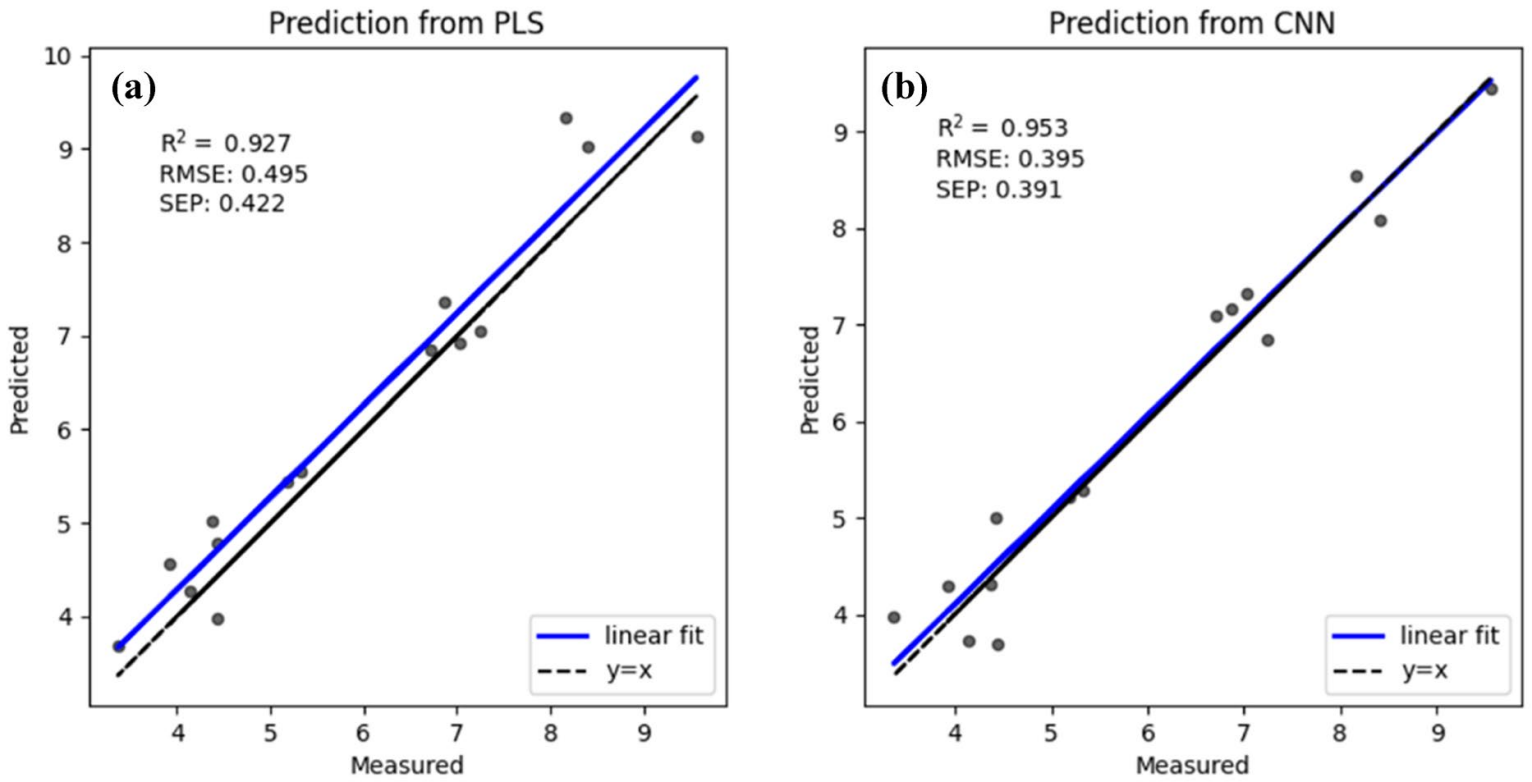
Comparison between predicted TOC concentrations and the true TOC concentrations constructed with the PLS and CNN models

To evaluate the accuracy of the PLS and CNN models, Fig. 5 shows the regression results of the measured and predicted values of TOC with the PLS and CNN models. The model performance was evaluated with RMSE and R^2^. For the PLS model result, the RMSE increases from 0.230 to 0.495, and the R^2^ result decreases from 0.975 to 0.927 between the training dataset and test dataset. There was only a slight decline in accuracy from the training dataset to the test dataset. Other variant models, such as iPLSR and siPLSR, may obtain more accurate results through specific wavelength selection. For the CNN model result, the RMSE increases from 0.182 to 0.395, and the R^2^ result decreases from 0.984 to 0.953 between the training and test datasets. The R^2^ values calculated with both models were better than 0.9, indicating that both models perform well. Because the samples were collected from the same place, a relatively accurate regression result is understandable, while different samples collected from more places may lead to a worse regression result.

**Fig. 6:**
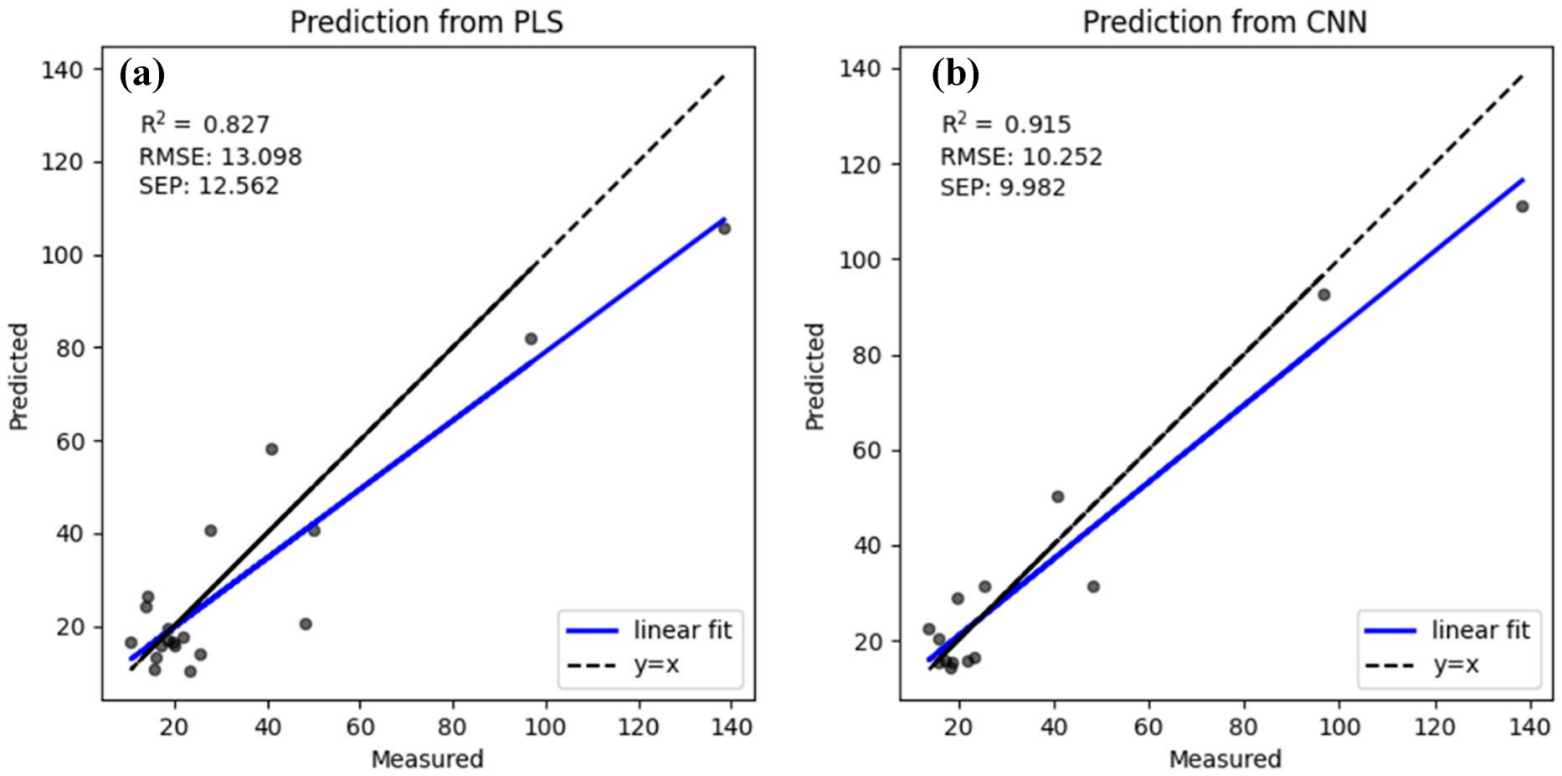
Comparison between predicted TSS concentrations and the true TSS concentrations constructed with the PLS and CNN models

Figure 6 shows the regression results of the measured and predicted values of TSS with the PLS and CNN models, and the model performance was evaluated with RMSE and R^2^. For the PLS model, the RMSE increases from 10.15 to 13.098, and the R^2^ result decreases from 0.846 to 0.827 between the training and test dataset. For the CNN model, the RMSE increases from 1.43 to 10.252, and the R^2^ results decrease from 0.997 to 0.915 between the training and test dataset. The R^2^ calculated with the PLS model was below 0.9 because the TSS values were located in a large range from 7.383 to 139.958. Accuracy may be improved with different models within different concentration ranges.

The experimental results of this study showed that both PLS and CNN models may obtain an accurate inversion result. CNN model performs better than PLS with RMSE and R^2^ result. If the samples are acquired in different places and different water types, the inversion algorithm should include a step to classify the water types and different water types may use a specific model. The application demonstrated that UV–Vis spectroscopy is a feasible way to monitor water parameter online and in situ, especially when the intensive monitoring of water resources is demanded. Since water use on a global scale has exceeded twice that of population growth and water stress is becoming serious because water resource is unevenly distributed, urbanization and contamination have restricted the usage of water resource. Most of the agricultural catchments is located in small towns near small rivers and lakes where there is not enough infrastructure to monitor the water quality. This method is nearly in real time and may allow quickly decisions regarding the quality of water without chemical waste generation. The technology is suitable to monitor the water quality of the agricultural catchment because this area has not received adequate attention. When the monitoring and sampling equipment is placed close to the confluence of the river, UV–Vis spectroscopy with CNN model is capable of measuring water parameter nearly in real time. The proposed model is able to simplify all the measurement process and mathematical calculation, reducing the computation time, but not to reduce the analytical accuracy.

The CNN model developed in this work is created using turbidity compensated absorbance spectra as input dataset, one convolutional layer, one pooling layer and one fully connected layer to output the water parameter. This CNN benchmark architecture is similar to other previously reported study for water parameter inversion. Studies have found the appropriate number of neurons and nodes in the network structure. To increase the computation capability and nonlinear capacity of the network, it is directly to increase the number of the neuros and nodes. When the number is large, the model performs well and eliminates the noise in the training samples. However, this CNN model still has some disadvantages regarding the time-consuming and possible overfitting.

Irrigation plays an import role in food security and economic development in many countries. Agriculture in developed countries has dramatically increased the efficiency with the use of animal breeding and fertilizers. The aim of farming system is for feeding local populations and profit instead of environmental protection in agricultural catchment, resulting in losses of nutrients and sediment which is the key indicator of poor water quality. There is a severity of water quality impairment on surface and ground waters with the intensification of production systems. However, the water quality of the agricultural catchment has not received effective monitoring. With monitoring and sampling equipment placed close to the confluence with the river, a UV–Vis spectrometry water probe can be used to monitor water quality with CNN model effectively nearly in real time. Thus, the technology may help to draw up strategies whose aim is to maintain healthy water quality while buffering economic change.

## Conclusion

In the present study, the efficiency of the PLS and CNN models was investigated in the prediction of two water quality parameters, TSS and TOC, in a river confluence near Sherfield-on-Loddon. The performance of the models was evaluated using RMSE and R^2^. The results indicated that the CNN model with minimum parameters could be successfully used for predicting TSS and TOC concentrations. It was found that in this study, the PLS and CNN models may show comparable results, but the CNN model was much more accurate (for example, RMSE = 13.098 mg/L for the PLS model in contrast to RMSE = 10.252 mg/L for the CNN model). In both models, predictions of TOC concentration were found to be better than those of TSS. The results in this study suggest that UV–Vis spectroscopy, in combination with the PLS and CNN models, provides an accurate performance to estimate water parameters online. The implication of this work verifies the potential possibility of using portable UV–Vis spectroscopy connected to a computer to predict the water parameter in places where there is no required infrastructure with a conventional method. Once the technological process is established, there is no need for sample preparation and chemical reagents, reducing the chemical waste and analysis time. Furthermore, if the computer can send spectrum data with the network to a central controller that can execute model calculations, water parameters can be monitored online in real time.

## Data Availability

The datasets generated and/or analyzed during the current study are available from the corresponding author on reasonable request.

## Code availability

The code generated during the current study is available from the corresponding author on reasonable request.
